# Skeletal muscle pathology of infantile Pompe disease during long-term enzyme replacement therapy

**DOI:** 10.1186/1750-1172-8-90

**Published:** 2013-06-20

**Authors:** Sean N Prater, Trusha T Patel, Anne F Buckley, Hanna Mandel, Eugene Vlodavski, Suhrad G Banugaria, Erin J Feeney, Nina Raben, Priya S Kishnani

**Affiliations:** 1 Division of Medical Genetics, Department of Pediatrics, Duke University Medical Center, Durham, NC 27710, USA; 2 Department of Pathology, Duke University Medical Center, Durham, NC 27710, USA; 3 Metabolic Unit, Meyer Children’s Hospital, Rambam Medical Center, Technion Faculty of Medicine, Haifa, Israel; 4 Department of Pathology, Rambam Medical Center, Technion Faculty of Medicine, Haifa, Israel; 5 Laboratory of Muscle Stem Cells and Gene Regulation, National Institute of Arthritis and Musculoskeletal and Skin Diseases, National Institutes of Health, Bethesda, MD 20892, USA

**Keywords:** Pompe disease, Glycogen storage disease type II, Enzyme replacement therapy, rhGAA, Alglucosidase alfa, Myozyme, Skeletal muscle, Pathology, Autophagy, Genetic diseases

## Abstract

**Background:**

Pompe disease is an autosomal recessive metabolic neuromuscular disorder caused by a deficiency of the lysosomal enzyme acid alpha-glucosidase (GAA). It has long been believed that the underlying pathology leading to tissue damage is caused by the enlargement and rupture of glycogen-filled lysosomes. Recent studies have also implicated autophagy, an intracellular lysosome-dependent degradation system, in the disease pathogenesis. In this study, we characterize the long-term impact of enzyme replacement therapy (ERT) with recombinant human GAA (rhGAA) on lysosomal glycogen accumulation and autophagy in some of the oldest survivors with classic infantile Pompe disease (IPD).

**Methods:**

Muscle biopsies from 8 [4 female, 4 male; 6 cross-reactive immunologic material (CRIM)-positive, 2 CRIM-negative] patients with a confirmed diagnosis of classic IPD were examined using standard histopathological approaches. In addition, muscle biopsies were evaluated by immunostaining for lysosomal marker (lysosomal-associated membrane protein-2; LAMP2), autophagosomal marker (microtubule-associated protein 1 light chain 3; LC3), and acid and alkaline ATPases. All patients received rhGAA by infusion at cumulative biweekly doses of 20–40 mg/kg.

**Results:**

Median age at diagnosis of classic IPD was 3.4 months (range: 0 to 6.5 months; n = 8). At the time of muscle biopsy, the patients’ ages ranged from 1 to 103 months and ERT duration ranged from 0 (i.e., baseline, pre-ERT) to 96 months. The response to therapy varied considerably among the patients: some patients demonstrated motor gains while others experienced deterioration of motor function, either with or without a period of initial clinical benefit. Skeletal muscle pathology included fiber destruction, lysosomal vacuolation, and autophagic abnormalities (i.e., buildup), particularly in fibers with minimal lysosomal enlargement. Overall, the pathology reflected clinical status.

**Conclusions:**

This is the first study to investigate the impact of rhGAA ERT on lysosomal glycogen accumulation and autophagic buildup in patients with classic IPD beyond 18 months of treatment. Our findings indicate that ERT does not fully halt or reverse the underlying skeletal muscle pathology in IPD. The best outcomes were observed in the two patients who began therapy early, namely at 0.5 and 1.1 months of age.

## Background

Pompe disease (OMIM 232300) is an autosomal recessive disorder caused by a deficiency of lysosomal acid alpha-glucosidase (GAA; OMIM 606800) leading to rapid glycogen accumulation in multiple tissue types, including skeletal, cardiac, and smooth muscle, and nervous tissue [1]. The clinical spectrum of Pompe disease varies broadly, with significant differences existing in age of onset, rate of disease progression, and overall clinical phenotype. Pompe disease is typically categorized into infantile and late-onset forms based on whether clinical symptoms develop prior to or beyond 1 year of age, respectively [2]. Infantile Pompe disease (IPD) is further subdivided into either classic or atypical (i.e., non-classic) forms based on the presence or absence of cardiomyopathy as a primary feature [3]. Given the availability of enzyme replacement therapy (ERT) with recombinant human GAA (rhGAA; alglucosidase alfa; Myozyme^®^; Genzyme Corp., Cambridge, MA), most IPD patients have experienced gains in overall survival and quality of life [4-7]. However, many previously unknown complications have emerged [8], and the impact of long-term ERT on the relative contribution of lysosomal and autophagic pathologies to skeletal muscle damage is not known.

In a study involving muscle biopsies performed in 8 IPD patients at baseline and at 3 and 12 months post-ERT with Chinese hamster ovary (CHO) cell-derived rhGAA, significant variability in overall glycogen clearance was observed [7,9]. It was also noted that those patients who started treatment at earlier ages and/or prior to severe motor impairment seemed to have better glycogen clearance [7,9]. In an earlier study, muscle biopsies obtained from 4 IPD patients treated for 18 months with rhGAA derived from rabbit milk showed variable yet appreciable improvements in skeletal muscle morphology with good correlation between clinical and histopathological findings [10]. A later study examining lysosomal and autophagic pathologies in patients with IPD or late-onset Pompe disease (LOPD) at baseline and 6 months after the initiation of ERT demonstrated that autophagic buildup becomes more prominent as the lysosomal pathology diminishes on therapy [11]. However, no systematic muscle pathology studies have been conducted beyond 18 months on ERT, although some ERT-treated survivors with IPD are now over 13 years of age. These studies are of particular importance and relevance given that residual motor deficits have been documented in long-term survivors of IPD despite continued ERT [8,12]. Herein, we present the findings of muscle biopsies taken from a series of patients with classic IPD who have been on ERT for up to 96 months (8 years).

## Methods

### Patient cohort

Muscle biopsies from 8 (4 male, 4 female; 6 CRIM-positive, 2 CRIM-negative) patients with classic IPD (including 6 patients from Duke University Medical Center, Durham, NC, USA and 2 from Rambam Medical Center, Haifa, Israel) were obtained as part of clinical trials or concurrent with scheduled surgical procedures. Prior to biopsy, written informed consent was provided by the respective parents/guardians for all patients under institutional review board-approved protocols. All patients received rhGAA biweekly by infusion at cumulative doses of 20–40 mg/kg (U.S. Prescribing Information, Genzyme Corp., 2006), and the 2 CRIM-negative patients also received successful immune-tolerance induction (ITI) therapy [13]. In addition to histopathological and longitudinal clinical information, data regarding the cumulative dose of the drug versus time were collected for all patients. Age at diagnosis and ERT initiation for individual patients and the patient cohort are shown as raw values and values corrected to gestational age (CGA) at birth (i.e., corrected to 40 weeks gestational age if born prior to 40 weeks). Patients were followed through November 2012, at which time the database was locked.

### Tissue preparation, staining, and microscopy

Biopsy samples were processed for routine histology and LAMP2/LC3 staining. Hemaotoxylin and eosin (H&E) and periodic-acid Schiff diastase (PAS-D) staining were performed according to standard procedures. Glutaraldehyde-fixed tissue was used to prepare epon-embedded sections. ATPase staining was performed after acid and alkaline preincubations according to standard procedures. LAMP2/LC3 immunostaining was performed on isolated muscle fibers as previously described [14]. In many cases, muscle bundles (up to 20 fibers) were isolated instead of single fibers because of the condition of the tissue and the small fiber diameter in infantile patients. The following primary antibodies were used: anti-LC3 (1:250; provided by Dr. Takashi Ueno, Juntendo University School of Medicine, Japan) and mouse anti-human LAMP2 monoclonal antibody (1:100; BD Biosciences Pharmingen, San Diego, CA). Alexa Fluor^®^ 488 and 568 secondary antibodies were purchased from Invitrogen™ (Carlsbad, CA). For each patient, approximately 100 fibers were analyzed by confocal microscopy (Zeiss LSM 510 META), and the number of fibers with autophagic pathology was counted.

## Results

Histopathological analyses of skeletal muscle tissue were performed in 8 (4 male, 4 female; 6 CRIM-positive, 2 CRIM-negative) patients with classic IPD. Demographic and mutation data for these patients are summarized in Table 1. Median age at confirmed IPD diagnosis was 3.4 months (range: 0 to 6.5 months) [2.7 months CGA (range: -0.3 to 5.5 months)]. Median age at ERT initiation was 5.7 months (range 0.5 to 7.0 months) [5.5 months CGA (range: 0.2 to 6.0 months)].

**Table 1:** Baseline demographics, mutations, and clinico-pathological data for the 8 patients with classic infantile Pompe disease

**Patient ID CRIM status**	***GAA *mutations**	**Age at diagnosis (mo)**	**Age at ERT Start (mo)**	**Biweekly ERT Dose (mg/kg)**	**Biopsy site and age/CGA (mo)**	**Clinical outcome on ERT**^**§**^	**Autophagic pathology**
1. Male, Israeli Druze +	c.1210G > A (p.Asp404Asn)	0	1.1	20/40	Q, 1.0	Good response; only articulatory disorder remains after 103.9 mo.	Present in 2% of fibers (biopsy after 96 mo. of ERT)
					Q, 13.1		
	c.1064 T > C (p.Leu355Pro)						
					Q, 97.1		
2. Male, Israeli Druze +	c.1064 T > C (p.Leu355Pro) homozygous	4.0; 2.6 CGA	7.0; 5.6 CGA	20	Q, 6.7/ 5.3	Initial developmental progress; decline in muscle strength and function after 89 mo.	Prominent after 96 mo. of ERT; autofluorescent inclusions
					Q, 19.0/ 17.6		
					Q, 103.0/101.6		
3. Female, Hispanic +	c.1802C > T* (p.Ser601Leu)	0.2;−0.3 CGA	5.4; 4.9 CGA	20/40	Q, 5.3/4.8	Initial developmental progress and motor gains; decline after 56 mo.	Detected on PAS-stained sections after 12 mo. of ERT
					Q, 17.4/16.9		
	c.1099 T > C (p.Trp367Arg)						
					Q, 90.4/ 89.9		
4. Male, Caucasian +	c.525delT (p.Glu176ArgfsX45)	0;−0.3 CGA	0.5; 0.2 CGA	20	Q, 77.5/77.2	Good response; normal gross motor development, no hypernasal speech up to 81.1 mo.	Present in 60-70% of fibers (biopsy after 77 mo. of ERT); autofluorescent inclusions
	c.1642G > T^†^ (p.Val548Phe)						
5. Male, Caucasian +	c.1933G > A (p.Asp645Asn) homozygous	2.7	2.9	20/40	SCM, 85.9	Motor decline after 35 mo.; gastrostomy tube supplementation	Present in >10% of fibers (biopsies after 83–84 mo. of ERT)
					Q, 86.9		
6. Female, Caucasian +	c.655G > A (p.Gly219Arg) homozygous	6.5; 5.5 CGA	7.0; 6.0 CGA	20/40	Q, 56.0/55.0	Motor decline after 14 mo.; gastrostomy tube supplementation	Present in occasional fibers (biopsy after 49 mo. of ERT)
7. Female, African-Am. -	c.2560C > T (p.Arg854X) homozygous	5.5; 5.0 CGA	6.3; 5.8 CGA	20	Strap, 7.3/6.8	Limited motor gains, invasive ventilation and gastrojejunostomy tube; death at 21 mo. of age	Not identifiable due to severe muscle deterioration
					Q, 20.3/19.8		
8. Female, Hispanic -	c.1195-18_2190-20del (p.Asp399ValfsX6) homozygous	5.2	6.0	20/40^‡^	S, 24.0	Severe motor impairment; ventilator support and gastrojejunostomy tube	Not identifiable due to severe muscle deterioration

^*^Allele has two polymorphic sites: c.1726G > A (p.Gly576Ser), c.2065G > A (p.Glu689Lys).

^†^Allele contains c.1880C > T nucleotide change (p.Ser627Phe); function unknown.

^‡^Patient 8 received cumulative dose of 40 mg/kg (20 mg/kg weekly). The regimen was increased after one month of therapy.

^§^Cardiac improvement seen in all patients.

Q: quadriceps muscle.

SCM: sternocleidomastoid muscle.

Strap: anterior neck “strap” muscle.

S: soleus muscle.

+: CRIM-positive.

-: CRIM-negative.

**Figure 1:**
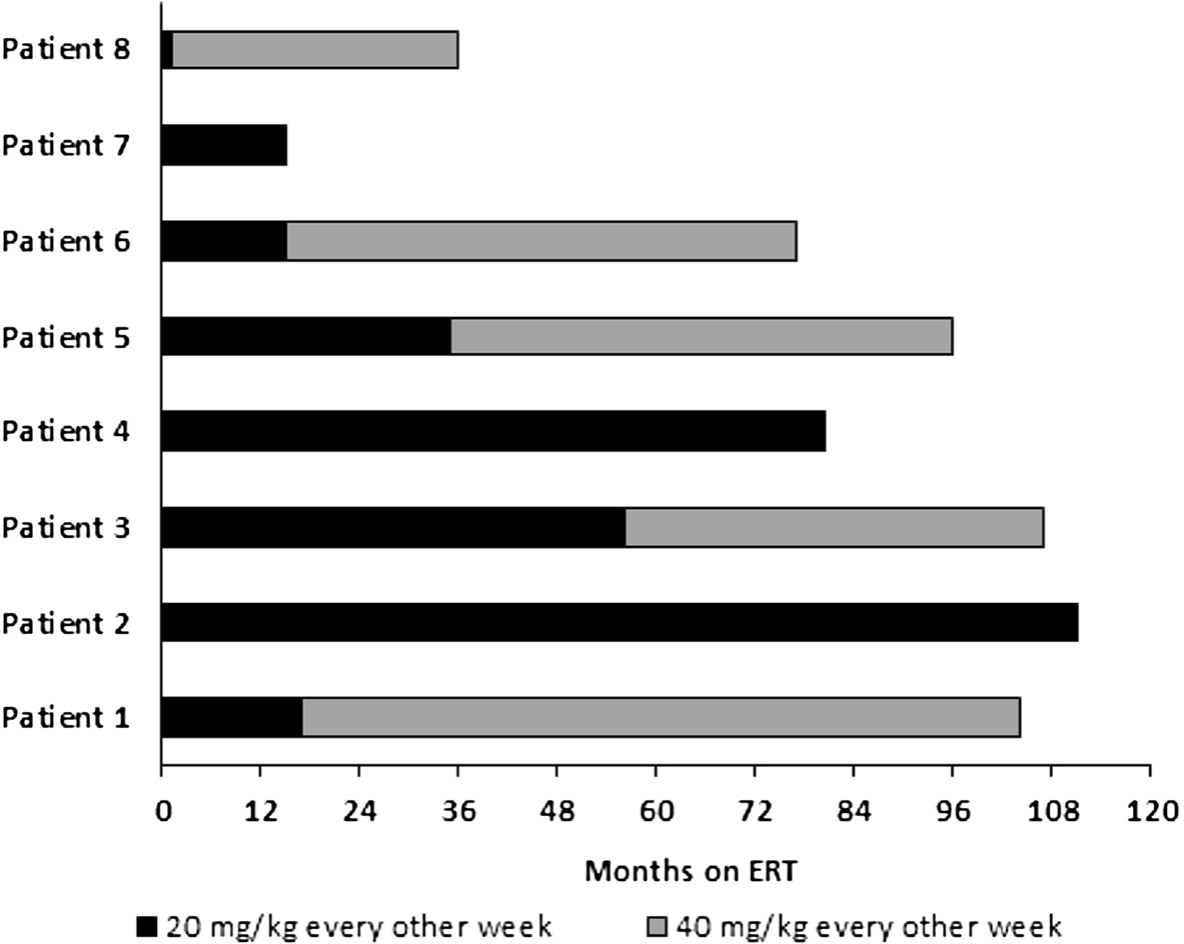
Cumulative biweekly dose (mg/kg) of alglucosidase alfa enzyme replacement therapy versus time (months) for the 8 patients with classic infantile Pompe disease.

Three of 8 patients who had been part of the original pivotal trials of rhGAA [6] had 3 biopsies each; two of 8 patients had 2 biopsies each, and the remaining three patients had a single biopsy each. Age at the time of biopsy ranged from 1.0 to 103.0 months, with time on ERT ranging from 0 (i.e., baseline/pre-ERT; n = 3) to 96 months. Patient-specific ERT dose information is shown in Figure 1. In all the cases examined, no correlation between the fiber type and the degree of muscle involvement was observed (not shown). Each patient’s clinical status and muscle pathology are presented below and in Table 1.

### Patient 1

Patient 1, an Israeli Druze male with an extensive family history of IPD, was diagnosed with CRIM-positive IPD soon after birth. ERT (40 mg/kg biweekly by infusion) was commenced as part of a clinical trial at the age of 1.1 months (also 1.1 months CGA; see patient N in [6]). Following 17 months of ERT, the patient’s dose was reduced to 20 mg/kg biweekly in accordance with the package insert (alglucosidase alfa; Myozyme^®^; U.S. Prescribing Information, 2006, Genzyme Corp., Cambridge, MA). Hypotonia was absent upon physical examination prior to the start of ERT. Baseline echocardiography demonstrated left ventricular hypertrophy (LVH); baseline left ventricular mass index (LVMI) was above normal at 59.31 g/m^2^ but normalized following ERT (week 78: 40.76 g/m^2^; week 104: 52.46 g/m^2^). The patient began to walk unassisted at the age of 12 months and remained within the normal range of developmental milestones for his age group. Currently, at the age of 8.7 years, he is strong and active. The only clinical sign of Pompe disease is an articulatory disorder with prominent nasal speech.

**Figure 2:**
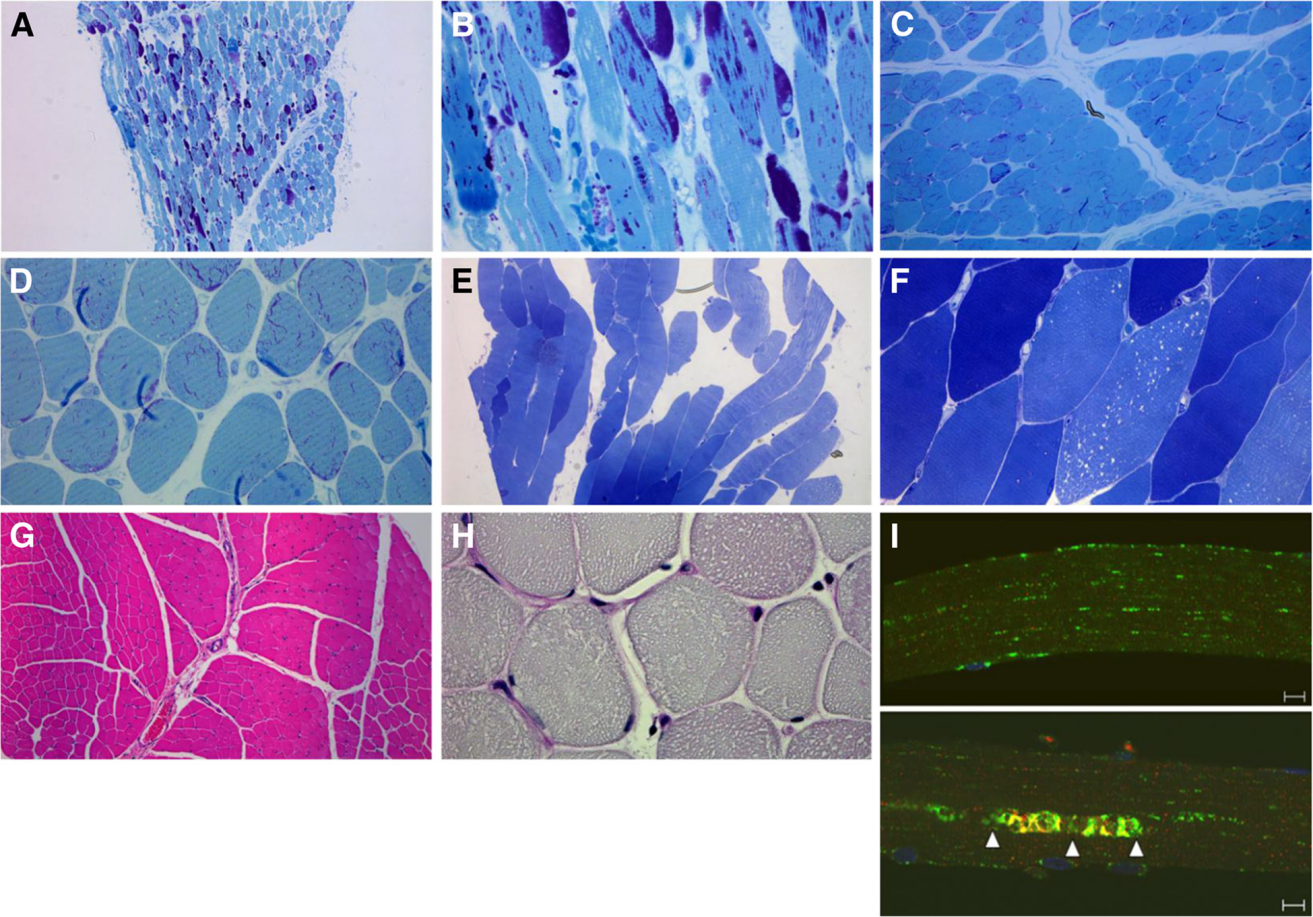
**Analysis of quadriceps muscle biopsy from Patient 1 prior to and following 12 and 96 months of ERT.** (**A**) Epon-embedded PAS/Richardson’s-stained section shows mild to extensive vacuolation in many fibers (magnification 25×). (**B**) At a higher magnification (200×), it is evident that nearly all fibers are at least mildly affected. (**C**, **D**) After 12 months of ERT (age 13.1 months), no effaced fibers are seen, and there is much less glycogen overall (100× and 200×, respectively). (**E**, **F**) After 96 months of ERT (age 97.1 months), there is only subtle microvacuolar damage in occasional fibers and no evident glycogen accumulation (100× and 630×, respectively). (**G**, **H**) H&E-stained paraffin and PAS-D sections (25× and 630×, respectively) show little interstitial stroma in otherwise normal-looking fibers. (**I**, top and bottom) LAMP2/LC3 staining (lysosomes: green; autophagosomes: red) demonstrates mostly intact muscle fibers (for example, top); autophagic pathology can be seen in ~2% of fibers (bottom; arrowheads). Bar: 10 μm.

Histologic examination of a quadriceps biopsy taken 2 days prior to ERT initiation (age 1.0 month) showed a spectrum of fiber involvement ranging from mild vacuolation to effacement (i.e., replacement of myocyte myofibrillar structures by visibly-evident glycogen within the boundary of the muscle cell); more than 10% of fibers were severely affected (Figure 2A). All fibers were at least minimally affected, but no obvious autophagic pathology was seen (Figure 2B).

In a quadriceps biopsy taken after 12 months of ERT (age 13.1 months), only rare fibers were effaced or severely vacuolated, and overall glycogen staining appeared to be substantially less than in the baseline biopsy (Figure 2C). However, affected fibers and increased interstitial stroma were seen at a higher magnification (Figure 2D). After 96 months of ERT (age 97.1 months), no glycogen accumulation was seen in most fibers (Figure 2E, F, G, H). LAMP2/LC3 staining showed mostly intact fibers without lysosomal pathology (Figure 2I, top), and only about 1/50 fibers contained autophagic buildup, which was limited to relatively small areas (not exceeding 100 μm) (Figure 2I, bottom). Altogether, the patient’s minimal lysosomal and autophagic pathologies parallel his favorable clinical response.

### Patient 2

Patient 2, an Israeli Druze male, was brought to the metabolic clinic at the age of 4 months; his parents were concerned that he might be suffering from the same “unknown” muscle disease that caused the death of his 3-year-old brother. On admission, the baby had truncal hypotonia (severe head lag) and reduced knee reflexes; levels of serum creatine phosphokinase (CPK), aspartate aminotransferase (AST), and alanine aminotransferase (ALT) were elevated. Baseline echocardiography demonstrated LVH (LVMI not available). Diagnosis of CRIM-positive IPD was confirmed, and ERT (20 mg/kg biweekly) was commenced at the age of 7.0 months (5.6 months CGA) as part of a clinical trial (see patient B in [6]). Cardiac parameters normalized 3 months following ERT initiation. The patient achieved unsupported sitting by the age of 9 months and took his first independent steps at 14 months of age. He was able to participate in sports with his peers from a young age. The only symptoms of Pompe disease were facial muscle weakness with abnormal articulation and hypernasal resonance, which were partially improved with speech therapy. Since 8 years of age, he has demonstrated clinical sequelae seen in long-term IPD survivors, including dysphagia (with episodes of choking during meals), muscle weakness, and severe muscle pain following activities he formerly performed with ease. Currently, at the age of 9.8 years, he experiences difficulty climbing stairs and presents with a subtle waddling gait following strenuous activity. His speech and phonation have also deteriorated recently.

Histologic examination of a right quadriceps biopsy taken 9 days prior to ERT (age 6.7 months, or 5.3 months CGA) initiation showed a spectrum of fiber involvement ranging from mild vacuolation to effacement; about 10% of fibers were severely affected, and none appeared entirely normal (Additional file 1: Figure S1A). There was some fibrosis but autophagic debris was not evident (Additional file 1: Figure S1B). In a biopsy of the right quadriceps taken after 12 months of ERT (age 19.0 months, or 17.6 months CGA), fewer fibers appeared effaced or severely vacuolated (Additional file 1: Figure S1C), and normal fibers were present (Additional file 1: Figure S1D). After 96 months of ERT (age 103.0 months, or 101.6 months CGA), a right quadriceps biopsy demonstrated marked variability in damage; some effaced fascicles, minimally affected fibers, and many normal fibers were seen (Additional file 1: Figure S1E and S1F, more affected areas, top; less affected areas, bottom). This was confirmed on H&E paraffin sections (Additional file 1: Figure S1G). PAS-D showed an increase in interstitial stroma, but fibrosis was not prominent even in severely-affected areas (Additional file 1: Figure S1H). Consistent with histologic data, LAMP2/LC3 immunostaining after 96 months of ERT showed variable degrees of fiber involvement: occasional fibers with grossly enlarged lysosomes, fibers with modest lysosomal expansion as well as normal looking fibers. A prominent feature in this biopsy was the presence of autophagic buildup in most fibers, which was missed on routine histologic examination; the buildup areas contained clearly discernible LC3-positive autophagosomes clustered in proximity to LAMP2-positive lysosomes (Additional file 1: Figure S1I). Furthermore, many autophagic areas contained autofluorescent balloon-like structures similar to those found in adult patients with Pompe disease [15]. Thus, in patient 2, a modest effect of ERT on lysosomal pathology and the emergence of autophagic buildup are likely responsible for the observed clinical deterioration.

### Patient 3

Patient 3, a Hispanic female with a family history of Pompe disease, was diagnosed with CRIM-positive IPD at the age of 0.2 months (−0.3 months CGA). ERT was initiated at the age of 5.4 months (4.9 months CGA) at 20 mg/kg biweekly. Due to motor decline after 56 consecutive months, the dose was increased to 40 mg/kg biweekly. Baseline echocardiography demonstrated LVH with LVMI of 266 g/m^2^; cardiac parameters normalized by 33 months of age (LVMI 49 g/m^2^, shortening fraction 41%). The patient achieved independent sitting by 7 months of age, started walking at 14 months of age, and has always fed by mouth; however, she has received supplementary overnight gastrostomy tube feeds since 7 years of age. Currently, at 9.4 years, the patient does not require invasive ventilation, has normal cardiac size and function, and can ambulate with the assistance of a walker. She has several features of the evolving phenotype of infantile survivors, including ptosis, lingual weakness, myopathic faces, scoliosis, frequent falls, and the need for ankle-foot orthoses [8]. Like patients 1 and 2, patient 3 also participated in the original pivotal trial of alglucosidase alfa (see patient H in [6]). Right quadriceps biopsies were collected at baseline and 12 months on ERT [7,9], and an additional left quadriceps biopsy was obtained at 91 months of age (85 months on ERT).

Histologic examination of a right quadriceps biopsy taken 2 days prior to ERT initiation (age 5.3 months, or 4.8 months CGA) showed a spectrum of fiber involvement ranging from mild vacuolation to effacement; however, only 10% of fibers were severely affected (Additional file 2: Figure S2A). There was minimal fibrosis, and no obvious autophagic debris was seen (Additional file 2: Figure S2B). In the biopsy taken after 12 months of ERT (age 17.4 months, or 16.9 months CGA), more than half of the fibers were effaced or severely vacuolated; the tissue was very fibrotic (Additional file 2: Figure S2C), and autophagic debris was seen in some fibers (Additional file 2: Figure S2D; circled). After 85 months of ERT (age 90.4 months, or 89.9 months CGA), the biopsy revealed complete loss of myofibrillar architecture in all muscle fibers (Additional file 2: Figure S2E and S2F). Thus, despite increased dose of ERT, this patient had severe muscle damage and several clinical sequelae of IPD.

### Patient 4

Patient 4, a Caucasian male with a family history of Pompe disease, was diagnosed prenatally (age 0 months, or −0.3 months CGA) with CRIM-positive IPD. He was started on ERT at a cumulative dose of 20 mg/kg biweekly at the age of 0.5 months (0.2 months CGA). Baseline echocardiography demonstrated mild LVH, with an LVMI of 70 g/m^2^; LVMI normalized by 7 months of age (LVMI: 48 g/m^2^). The patient had normal gross motor development on ERT, was able to sit independently by 6.5 months of age, and took his first unassisted steps at 16 months of age. He has never required invasive ventilator support and has always fed by mouth. At the age of 6.8 years he continues to do well, with independent ambulation, motor gains, and no hypernasal speech.

Histologic examination of a right quadriceps biopsy taken after 77 months of ERT (age 77.5 months, or 77.2 months CGA) showed mild pathology with many normal looking fibers (Additional file 3: Figure S3A), albeit with increased intramyofibrillar staining. Some fibers were vacuolated, varying from mild (diffuse vacuolation or 1–2 large vacuoles) to severely affected. Overall, less than 1% fibers were severely damaged or effaced. The proportion of affected fibers reached 30% in some fascicles. There was evidence of limited fiber regeneration (internal nuclei) in occasional fibers. PAS-D staining showed preservation of the myofibrillar architecture in most fibers and no obvious increase in interstitial stroma (Additional file 3: Figure S3B). Epon-embedded sections revealed relatively preserved fiber architecture; autophagic debris was detected in some fibers (Additional file 3: Figure S3C; circled). LAMP2/LC3 immunostaining showed autophagic buildup in 60-70% of fibers (Additional file 3: Figure S3D). In this case, autophagic pathology appears to be the major abnormality in skeletal muscle after 77 months of treatment. Autofluorescent inclusions, similar to those seen in fibers from patient 2, were detected in this patient’s biopsy (Additional file 3: Figure S3D)**.** Similar to patient 1, this individual commenced ERT at a very early age, and his clinical status compares favorably to the others.

### Patient 5

Patient 5, a Caucasian male, presented at the age of 2 months with an upper respiratory tract infection; he had marked cardiomegaly on a chest x-ray obtained to evaluate this condition. The patient was diagnosed with CRIM-positive IPD and began ERT at 2.9 months of age (also 2.9 months CGA) with a cumulative biweekly dose of 20 mg/kg for the first 35 months and 40 mg/kg subsequently due to motor decline. At baseline, marked LVH (LVMI of 239 g/m^2^ and EF 45%) was present. Significant improvement in cardiac function and size was seen early-on during therapy, with normalization of cardiac parameters by 12 months (LVMI: 41 g/m^2^). Swallowing difficulties necessitated gastrostomy tube supplementation for nutrition. He is currently 99 months (8 years, 3 months) old, has a normal LVMI, has never required invasive ventilation, continues to receive gastrostomy tube supplementation, and is able to walk independently although he has an abnormal gait and ankle contractures.

Histologic examination of a right quadriceps biopsy taken during port revision surgery after 84 months of ERT (age 86.9 months) showed striking variation in pathology between fascicles (Additional file 4: Figure S4A). There was evidence of fiber regeneration (internal nuclei) in the less damaged part of the biopsy. PAS-D staining demonstrated preservation of the myofibrillar architecture of some fibers and obliteration of the adjacent ones, with mild fibrosis (Additional file 4: Figure S4B, left and right). Epon-embedded sections and LAMP2/LC3 immunostaining confirmed this striking variability ranging from well-preserved fibers with minimal or no lysosomal pathology [10% of which contained autophagic buildup (not shown)] to severely affected fibers (Additional file 4: Figure S4C and S4D, respectively).

Similarly, histologic examination and LAMP2/LC3 immunostaining of a left sternocleidomastoid (SCM) biopsy after 83 months of ERT (age 85.9 months) showed prominent damage and variability in muscle pathology and autophagic buildup (Additional file 4: Figure S4E and S4F). There was evidence of fiber regeneration and focal reactive inflammation, Thus, despite increased dose of ERT, both neck and leg muscles showed significant pathology, which varied considerably within each muscle group; this pathology reflects the inadequate clinical response with several long-term sequelae in this patient.

### Patient 6

Patient 6, a Caucasian female, presented with failure to thrive and congestive heart failure. She was diagnosed with CRIM-positive IPD at the age of 6.5 months. ERT was started at 7.0 months (6.0 months CGA) at a cumulative biweekly dose of 20 mg/kg for the first 14 months and then 40 mg/kg subsequently due to clinical plateau. Baseline echocardiography demonstrated marked LVH (with LVMI of 224 g/m^2^), which essentially normalized by 22 months of age (LVMI: 64.9 g/m^2^). Right quadriceps muscle biopsy was obtained at 56 months of age (49 months on ERT) during bilateral hip contracture release. At the time of surgery, the patient was able to maintain quadruped independently for 1 minute or stand with support for a few seconds. The patient has always been orally fed with gastrostomy tube supplementation, but has never been ventilator dependent.

Histologic examination of a right quadriceps biopsy taken after 49 months of ERT (age 56.0 months, or 55.0 months CGA) showed severe skeletal muscle pathology. H&E-stained sections revealed replacement of the sarcoplasm of the majority of muscle fibers by hematoxophilic material; the severely damaged and relatively unaffected fibers were randomly distributed irrespective of fiber type (data not shown) (Additional file 5: Figure S5A). There was evidence of fiber regeneration (internal nuclei) in some of the intact fibers. PAS-D staining confirmed moderate fibrosis (Additional file 5: Figure S5B) and highlighted the dichotomy of damage with preservation of the myofibrillar architecture of some fibers and obliteration of adjacent ones. Epon-embedded sections and LAMP2/LC3 immunostaining confirmed this heterogeneity (Additional file 5: Figure S5C and S5D). There was fibrosis around both the damaged and undamaged fibers (Additional file 5: Figure S5C). Autophagic debris was not seen on bright-field microscopy, but was detected by LAMP/LC3 staining in occasional fibers (Additional file 5: Figure S5D; bottom fiber). In this case, it appears that even the initial response to ERT was less dramatic than in other CRIM-positive patients. The patient’s underlying muscle histopathology correlates well with the limited improvement in motor function.

### Patient 7

Patient 7, an African American female*,* presented with decreased muscle tone at birth, feeding difficulties and weakness of extremities at 2 months, and persistent cardiomegaly and failure to thrive by the age of 5 months. The patient was diagnosed with CRIM-negative IPD at 5.5 months (5.0 months CGA) and began ERT (20 mg/kg rhGAA biweekly) plus immunomodulation with methotrexate and rituximab ([13]) at 6.3 months (5.8 months CGA). Baseline echocardiography demonstrated marked left ventricular hypertrophy (LVMI of 351.4 g/m^2^). An anterior neck “strap” muscle biopsy was obtained during tracheostomy tube placement at 7.3 months of age. A marked improvement in LVH (LVMI: 60.6 g/m^2^) was observed at the age of 21 months, and the patient experienced limited motor gains such as improved head control and increased ability to move extremities against gravity. However, the patient was never able to achieve independent sitting or full head control and continued to require invasive ventilation and gastrojejunostomy tube placement throughout the course of ERT. She died unexpectedly at the age of 21 months of a presumed cardiac event.

**Figure 3:**
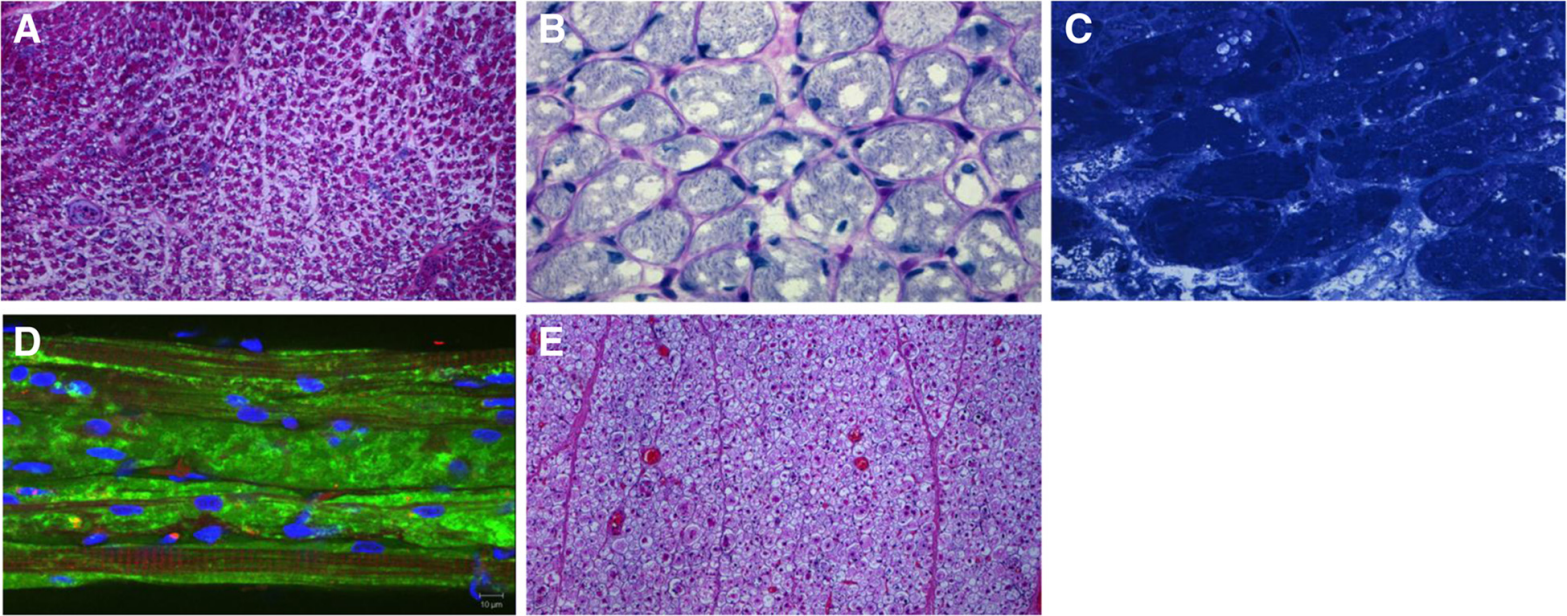
**Analysis of muscle biopsies from Patient 7 after 1 and 14 months of ERT. A-D**: Anterior neck “strap” muscle biopsy taken after 1 month of ERT (age 7.3 months, or 6.8 months CGA). (**A**) H&E-stained frozen section shows the extent of damage and fibrosis: vacuolation of more than 90% of myocytes; complete loss of myofibrillar architecture in 10% of myocytes (magnification 25×). (**B**) PAS-D staining demonstrates vacuolation of most fibers and increased interstitial stroma; however, much of the internal fiber architecture is preserved (630×). (**C**) Epon-embedded toluidine blue-stained section highlights the partial replacement of sarcoplasm by vesicular structures (630×). (**D**) LAMP2/LC3 immunostaining demonstrates extensive damage with only partial preservation of muscle structure (i.e., striations in the top two and bottom fibers); bright green staining, particularly in the middle fiber, likely indicates the presence of remnants of lysosomal membranes. Bar: 10 μm. (**E**) H&E-stained section of post-treatment quadriceps muscle from autopsy (i.e., after 14 months of ERT; age 20.3 months, or 19.8 months CGA) shows extensive damage, which appears to be more severe than that in the pre-ERT biopsy (25×).

Histologic examination of an anterior neck “strap” biopsy taken after 1 month of ERT (age 7.3 months, or 6.8 months CGA) showed widespread skeletal muscle pathology and vacuolization of most fibers. In H&E stained sections, the myofibrillar architecture was only partially preserved in about 80% of fibers; 10% appeared obliterated, and another 10% were relatively unaffected at this resolution (Figure 3A). PAS-D staining showed diffuse mild fibrosis, and preservation of much of the internal architecture in most fibers (Figure 3B). Epon-embedded, toluidine blue-stained sections revealed multiple vesicular structures (Figure 3C), but no autophagic debris. LAMP2/LC3 staining demonstrated extensive damage with only partial preservation of muscle structure in some fibers (Figure 3D). On autopsy (following 14 months of ERT), excess glycogen was found in all tissues examined. A sample of left quadriceps muscle (age 20.3 months, or 19.8 months CGA) showed more severe and diffuse damage than had been seen in the “strap” muscle biopsy taken 13 months before; now there was vacuolization of nearly all fibers and obliteration of the myofibrillar architecture in more than 95% of muscle cells (Figure 3E). Thus, despite successful ITI, the patient did not survive beyond 21 months of age. Correspondingly, the pathology showed profound muscle destruction.

### Patient 8

Patient 8, a Hispanic female, was found to have supraventricular tachycardia and ventricular hypertrophy at birth. She was diagnosed with CRIM-negative IPD at the age of 5.2 months (also 5.2 months CGA) and received biweekly infusions of ERT (20 mg/kg biweekly) with immunomodulation treatment at the age of 6.0 months (also 6.0 months CGA). After 4 weeks, the frequency of ERT was increased to weekly infusions because of severe hemidiaphragmatic weakness and hypotonia. A right soleus muscle biopsy was obtained during a bilateral Achilles tendon lengthening procedure at 24 months of age (18 months of ERT). At the time of biopsy, the patient exhibited severe motor impairments, including incomplete head control and inability to sit independently, maintain quadruped, creep or crawl. Although the patient continued to remain ventilator dependent and rely on gastrojejunostomy tube feeds throughout the course of ERT, improvement in hypertrophic cardiomyopathy was noted. Baseline echocardiogram showed severe biventricular hypertrophy (LVMI of 318.4 g/m^2^ and shortening fraction of 26.9%), but on the follow-up at 24 months of age these parameters became 72.5 g/m^2^ and 28.3%, respectively.

Histologic examination of a right soleus biopsy taken after 18 months of ERT (age 24.0 months) showed severe, diffuse skeletal muscle pathology. H&E-stained sections revealed vacuolization of nearly all muscle fibers and extensive loss of the myofibrillar architecture in most fibers (Additional file 6: Figure S6A). There was mild diffuse fibrosis with only lace-like remnants of myofibrillar structure (Additional file 6: Figure S6B). Both epon-embedded, toluidine blue-stained sections (S6C) and LAMP2/LC3 immunostaining (S6D) showed complete destruction of myofibers. Similar to patient 7, this CRIM-negative infant exhibited significant muscle damage at baseline and showed a poor response to ERT (despite successful ITI) both clinically and in terms of tissue morphology.

## Discussion

The aim of the present study was to examine histological findings and clinical symptoms of patients with classic IPD who had been on ERT for years. Previous studies examining the effects of ERT on skeletal muscle pathology had been limited to 72 weeks of treatment. However, the availability of therapy has led to the emergence of what appears to be a “new nosological entity” - skeletal muscle myopathy (combined with a series of additional symptoms) in long-term survivors of IPD. These survivors are a testament to both the successes and limitations of ERT: a dramatic improvement in cardiac function and prevention of cardiac failure, yet in many cases only a modest or negligible effect on skeletal muscle.

One of the questions we tried to address was the utility of muscle biopsy as a prognostic indicator of the patients’ response to ERT. In the three cases for which the pre-ERT (baseline) biopsies were available (patients 1, 2, and 3), histological findings appeared similar – variable degree of fiber involvement, approximately 10% severely damaged fibers, and no detectable autophagic abnormality. However, the clinical outcome is very different – patient 1 responded remarkably well, patient 2 began to deteriorate at age 8, and patient 3 showed a gradual progression of the disease. On the other hand, in each of the three cases, the findings on follow-up biopsies seem to correlate well with the clinical data – mostly normal histology with minor autophagic abnormalities in patient 1, significant skeletal muscle pathology in patient 2, and profound muscle destruction in patient 3. The same correlation was observed in patients 5, 6, 7, and 8, whose biopsies were available at different ages on ERT – a variable combination of lysosomal and autophagic pathologies in severely clinically affected children. Thus, although the prognostic value of initial muscle biopsy is questionable, muscle pathology in follow-up biopsies reflected clinical response. In none of the cases examined did we observe a normal or near normal biopsy in a patient who was significantly clinically affected. The reverse is also largely true – mildly symptomatic patients did not exhibit severe pathology. A possible exception is patient 4, who continues to do well on ERT at 6.8 years despite a prominent autophagic pathology in most fibers. It remains unclear whether autophagic buildup in this patient would eventually lead to clinical decline as seems to be the case in patient 2.

Despite the limitations of muscle biopsy, including great variability even in different regions of a single biopsy, it is still useful for monitoring disease severity and response to ERT. However, the inevitably invasive nature of muscle biopsies hampers longitudinal follow-ups from the same muscle group in the same patients over time. Furthermore, coordination of muscle biopsies with scheduled surgical procedures renders the site and time of biopsy entirely dependent on the site and time of surgery. Thus, consistent sampling from one particular muscle group, as would have been ideal, could not be achieved. Other modalities by which the disease severity can be monitored are currently being implemented. The urinary hexose tetrasaccharide (Hex_4_) biomarker that is used for overall assessment of disease severity and response to ERT [16,17] does not allow for the evaluation of the extent of involvement of particular muscle groups. Recent work has indicated the potential utility of whole-body magnetic resonance imaging (MRI) [18,19]. However, this technique has not yet been systematically evaluated for its ability to measure subtle changes in muscle pathology and will require further assessment of its diagnostic and prognostic potential in Pompe disease.

Although autophagic accumulation can be detected by routine histological examination of muscle biopsies, particularly when many fibers are affected (see patients 3 and 4), confocal microscopy and immunostaining of single fibers remains the best way to evaluate autophagic pathology. Consistent with our previous observations [11], autophagic abnormalities were negligible in baseline biopsies (patients 1, 2, and 3), but became prominent with prolonged ERT. Interestingly, autophagic buildup, often located in the core of muscle fibers, is usually seen in well-preserved fibers with minimal or no lysosomal pathology outside the buildup area (patients 1, 2, 4, 5, and 6), suggesting that these fibers responded to therapy reasonably well. In contrast to previous observations in both humans and mice [9,20-22], we did not see any appreciable difference in the involvement or response to ERT between type I and type II muscles.

In addition to lysosomal/autophagosomal vesicles (or remnants of these vesicles), the area of buildup often contains globular autofluorescent material, which is most striking in biopsies from patients 2 and 4 in this series. These structures are reminiscent of the acid phosphatase-positive inclusions detected in adult-onset Pompe patients, whose muscle pathology showed no typical vacuolated fibers [15]. The origin of these inclusions is not clear, but their presence in diseased human muscle cells points to yet another previously unrecognized pathology.

Several patients in this study (patients 2, 3, 5, and 6) initially showed clinical improvements on therapy, but they began to deteriorate at different time points ranging from 14 months in patient 6 to 8 years in patient 2, prompting the increase in the drug dosage to 40 mg/kg every 2 weeks (cumulative dose) in patients 3, 5, and 6. Although there are significant individual differences in the rates of motor deterioration, this clinical course – a decline following initial improvement – appears to be an emerging pattern in IPD patients on ERT, suggesting that even at extremely high doses of the drug, the therapy does not halt the progression of the disease**.** The reported initial decrease and eventual increase in Hex_4_ levels in patients on therapy [16] seem to mirror the clinical course, suggesting that indeed the replacement enzyme does not keep up with the rate of glycogen accumulation, likely due to inefficient delivery. Of note, we have found that all patients had slightly increased interstitial collagen and some degree of fibrosis, which may exacerbate the problem of inefficient delivery of the drug to skeletal muscle cells. Furthermore, as these patients continue to grow, they develop previously unrecognized complications of Pompe disease, such as ptosis, hypernasal speech, osteopenia, sensorineural and/or conductive hearing loss and gastroesophageal reflux [8]. Therefore, research into more effective second generation drugs with enhanced targeting properties is currently being pursued [23-25].

The pace of disease progression and the rate of clinical decline vary significantly even among patients with classic IPD. Although it is not currently feasible to accurately predict the course, CRIM-negative patients represent the most vulnerable group. In the two CRIM-negative patients in this study (patients 7 and 8), the drug failed to keep pace with the relentless disease progression despite successful immune modulation and the removal of high or sustained anti-rhGAA IgG titers [26-28].

Finally, it has been shown that muscle pathology in IPD patients develops *in utero*, and asymptomatic newborns may already have a significant number of severely affected fibers (see baseline biopsy of patient 1) [29-32]. There is a general consensus about the need to start therapy early before irreversible changes occur (indeed, the profoundly destroyed muscle fibers seen in the latest biopsies from patients 3, 6, 7, and 8, reached a “point of no return” and are unlikely to be rescued). Unfortunately, there is no clear-cut definition of what exactly “early” means. All CRIM-positive patients in this study were ventilator-independent and began therapy within 7 months of age, which is traditionally considered to be an “early” start. However, the outcome is suboptimal in many cases. Our data suggest that we should re-think the treatment paradigm for IPD and start considering “early” in terms of days or weeks rather than months. Indeed, the two patients in this study who responded best to therapy, patients 1 and 4, began therapy earlier than others – at five and two weeks, respectively; importantly, these two individuals were free from most of the secondary symptoms seen in IPD patients on therapy. An early start of therapy can be better achieved with the establishment of a newborn screening program; our results and those obtained in Taiwanese patients [33,34] strongly argue in favor of such a program. A long-term follow-up of patients identified by newborn screening would be of the utmost importance.

## Conclusions

This is the first report on the impact of rhGAA ERT on lysosomal glycogen accumulation and autophagic buildup in some of the oldest survivors of classic IPD. Despite long-term and regular administration of ERT, clinical and histological evidence from this study show that muscle damage persists and may increase in many patients. The balance between glycogen transport to lysosomes and its degradation by the drug tips in favor of lysosomal accumulation of the substrate as patients with IPD become older. Autophagic pathology emerges on ERT, particularly in fibers with little lysosomal abnormality. The best outcomes were observed in patients who began therapy the earliest, emphasizing the apparent benefits of newborn screening. The ethical aspects of such screening remain open to consideration.

## Abbreviations

ALT: Aspartate aminotransferase; AST: Alanine aminotransferase; BSA: Bovine serum albumin; CGA: Corrected gestational age; CHO: Chinese hamster ovary; CPK: Creatine phosphokinase; CRIM: Cross-reactive immunologic material; ERT: Enzyme replacement therapy; GAA: Acid alpha-glucosidase; H&E: Hematoxylin and eosin; Hex4: Hexose tetrasaccharide; IPD: Infantile Pompe disease; ITI: Immune tolerance induction; LAMP2: Lysosomal-associated membrane protein 2; LC3: Microtubule-associated protein 1 light chain 3; LOPD: Late-onset Pompe disease; LVH: Left ventricular hypertrophy; LVMI: Left ventricular mass index; MRI: Magnetic resonance imaging; PAS-D: Periodic acid–Schiff stain after diastase digestion; PBS: Phosphate buffered saline; rhGAA: Recombinant human acid alpha-glucosidase; SCM: Sternocleidomastoid muscle.

## Competing interests

The authors declare that they have no competing interests.

PSK reports receiving research and grant support from Genzyme. PSK also receives honoraria and consulting fees from Genzyme and is a member of the Pompe disease and the Gaucher Disease Registry Advisory Boards.

Duke University and the inventors of the method of treatment and precursors of the cell lines used to generate the enzyme (rhGAA) used commercially have received royalties pursuant to the University’s policy on inventions, patents, and technology transfer. This potential conflict for Duke University has been resolved through monetization.

## Authors’ contributions

SNP assisted in the design of the project, coordinated data collection, interpreted data, and helped draft and revise the manuscript. TTP assisted in the design of the project, coordinated data collection, interpreted data, and helped draft and revise the manuscript. AFB assisted in the design of the project, reviewed the light microscopic pathology of all cases, provided histopathologic interpretations for figure legends, contributed to data analysis and interpretation, and contributed to the text of the manuscript. HM contributed data and information on two cases in this manuscript. EV performed light and electron microscopy studies of patients 1 and 2 and provided the histopathological interpretations for these studies. SGB helped with data collection and revision of the manuscript. EJF analyzed data and participated in writing and preparation of the manuscript. NR generated data on autophagy, analyzed and interpreted data, and contributed to the writing of the paper. PSK contributed toward this project by helping conceive the project, by supervising the project and providing critical professional insights, and by helping write and revise the manuscript. All authors have seen and approved the final manuscript.

## Supplementary Material

Additional file 1: Figure S1 Analysis of quadriceps muscle biopsy from Patient 2 prior to and following 12 and 96 months of ERT. (A) Epon-embedded PAS/Richardson’s-stained section shows mild to extensive vacuolation in many fibers (magnification 25×). (B) At a higher magnification (630×), it is evident that nearly all fibers are at least mildly affected. There is evidence of fibrosis and myophagocytosis. (C, D) After 12 months of ERT (age 19.0 months, or 17.6 months CGA), fewer fibers are effaced overall, and normal looking fibers can be seen (25× and 630×, respectively). (E) After 96 months of ERT (age 103.0 months, or 101.6 months CGA), some sections show regions with effaced or prominently vacuolated fibers (top), whereas other sections show mildly affected and normal looking fibers (bottom) (25×). Similar observations are noted at a higher magnification (F, top and bottom; 100× and 630×, respectively). The data are confirmed by H&E (G; 25×) and PASD (H, top and bottom; 630×) staining. There is little interstitial fibrosis even in the more affected areas (H, top; H, bottom: less affected area). (I) LAMP2/LC3 staining demonstrates prominent autophagic buildup (top fiber; arrowheads) in many fibers, rare fibers with large expanded lysosomes (middle; arrows), and fibers with autofluorescent inclusions (bottom, open arrowheads). Bar: 10 μm. Click here for file

Additional file 2: Figure S2 Analysis of quadriceps muscle biopsy from Patient 3 prior to and following 12 and 85 months of ERT. (A) Epon-embedded toluidine blue/PAS-stained section shows mild to extensive vacuolation (magnification 25×). (B) At a higher magnification (100×), it is evident that nearly all fibers are at least mildly affected. There is minimal fibrosis. (C, D) After 12 months of ERT (age 17.4 months, or 16.9 months CGA), more fibers are effaced overall and there is severe fibrosis (C; 25×); autophagic debris (C) is detected at a higher magnification (D; circled; 630×). (E) After 85 months of ERT (age 90.4 months, or 89.9 months CGA), H&E-stained frozen sections show complete effacement of myofibrillar architecture (50×). (F) LAMP2/LC3 immunostaining demonstrates near‒complete destruction of muscle fibers. Bar: 10 μm. Click here for file

Additional file 3: Figure S3 Analysis of quadriceps muscle biopsy from Patient 4 after 77 months of ERT (age 77.5 months, or 77.2 months CGA). (A) H&E-stained frozen section shows the presence of vacuolated fibers in some fascicles (magnification 25×). Overall, muscle fibers are largely intact with limited evidence of regeneration (i.e., internal nuclei). (B) PAS-D staining demonstrates intact internal fiber architecture in most fibers, and no significant increase in interstitial stroma (630×). (C) Epon-embedded toluidine blue-stained section shows autophagic debris in mildly-affected fibers (circled; 630×). (D) LAMP2/LC3 immunostaining demonstrates largely intact muscle fibers (for example, top fiber in top panel) interspersed with moderately affected muscle fibers with prominent autophagic pathology (arrowheads). Bar: 10 μm. Click here for file

Additional file 4: Figure S4 Analysis of muscle biopsies from Patient 5 after 83 and 84 months of ERT. (A) H&E-stained frozen section shows the variation in pathology within a quadriceps biopsy: badly damaged fascicles are seen adjacent to largely intact ones; there is some evidence of regeneration (i.e., internal nuclei) (magnification 25×). (B, left and right) PAS-D staining demonstrates relatively intact fibers next to completely effaced ones; interstitial stroma are increased (630×). (C) Epon-embedded toluidine blue-stained section (630×) shows vacuolization in many fibers. (D, E) LAMP2/LC3 immunostaining demonstrates great variability of muscle fiber involvement in biopsies from both quadriceps (D) and SCM (E) muscles: a normal fiber (D; top fiber) next to a completely destroyed one (D; bottom fiber), a fiber with autophagic buildup (the buildup is seen in ~10 % of fibers) (E; arrowheads), and fibers with largely expanded lysosomes (E; arrows). Bar: 10 μm. (F; 25×) H&E-stained paraffin section of SCM muscle shows variation in pathology: severely damaged fascicles adjacent to less affected fascicles; there is some evidence of regeneration (i.e., internal nuclei); note the focal reactive mononuclear inflammation (lower right). Note: The images in A-D show tissue from the quadriceps biopsy taken after 84 months of ERT (age 86.9 months). The images in E and F show tissue from the SCM biopsy taken after 83 months of ERT (age 85.9 months). Click here for file

Additional file 5: Figure S5 Analysis of quadriceps muscle biopsy from Patient 6 after 49 months of ERT (age 56.0 months, or 55.0 months CGA). (A) H&E-stained frozen section shows the pattern of damage and fibrosis: vacuolization of more than 50% of myocytes, interspersed with fibers showing intact myofibrillar architecture and some internal nuclei indicating regeneration (magnification 25×). (B) PAS-D staining demonstrates relatively intact fibers in close proximity to completely effaced ones and increased interstitial stroma (630×). (C) Epon-embedded toluidine blue-stained section confirms the results obtained with H&E and PAS-D staining (630×). (D) LAMP2/LC3 immunostaining further demonstrates variability of muscle fiber involvement: a completely destroyed fiber (top fiber), a relatively preserved fiber with moderately enlarged lysosomes (middle fiber), and a fiber with autophagic accumulation (bottom fiber, arrowheads). Bar: 10 μm. Click here for file

Additional file 6: Figure S6 Analysis of soleus muscle biopsy from Patient 8 after 18 months of ERT (age 24.0 months). (A) H&E-stained frozen section shows the extent of damage and fibrosis: severe, diffuse vacuolization of more than 95% of myocytes (magnification 25×). (B) PAS-D staining demonstrates loss of internal muscle architecture in virtually all fibers (only lace-like remnants can be seen); there is an increase in interstitial stroma (630×). (C) Epon-embedded toluidine blue-stained section highlights the obliteration of sarcoplasm by small vesicular structures (630×). (D) LAMP2/LC3 immunostaining demonstrates complete destruction of myofibers with a few identifiable autophagosomes (arrows) in the top fiber. Bar: 10 μm. Click here for file
