# Supplementary figures and images for: Skeletal muscle pathology of infantile Pompe disease during long-term enzyme replacement therapy
Source: Orphanet J Rare Dis. 2013 Jun 20;8:90. doi: 10.1186/1750-1172-8-90 (PMC3691834; doi:10.1186/1750-1172-8-90)

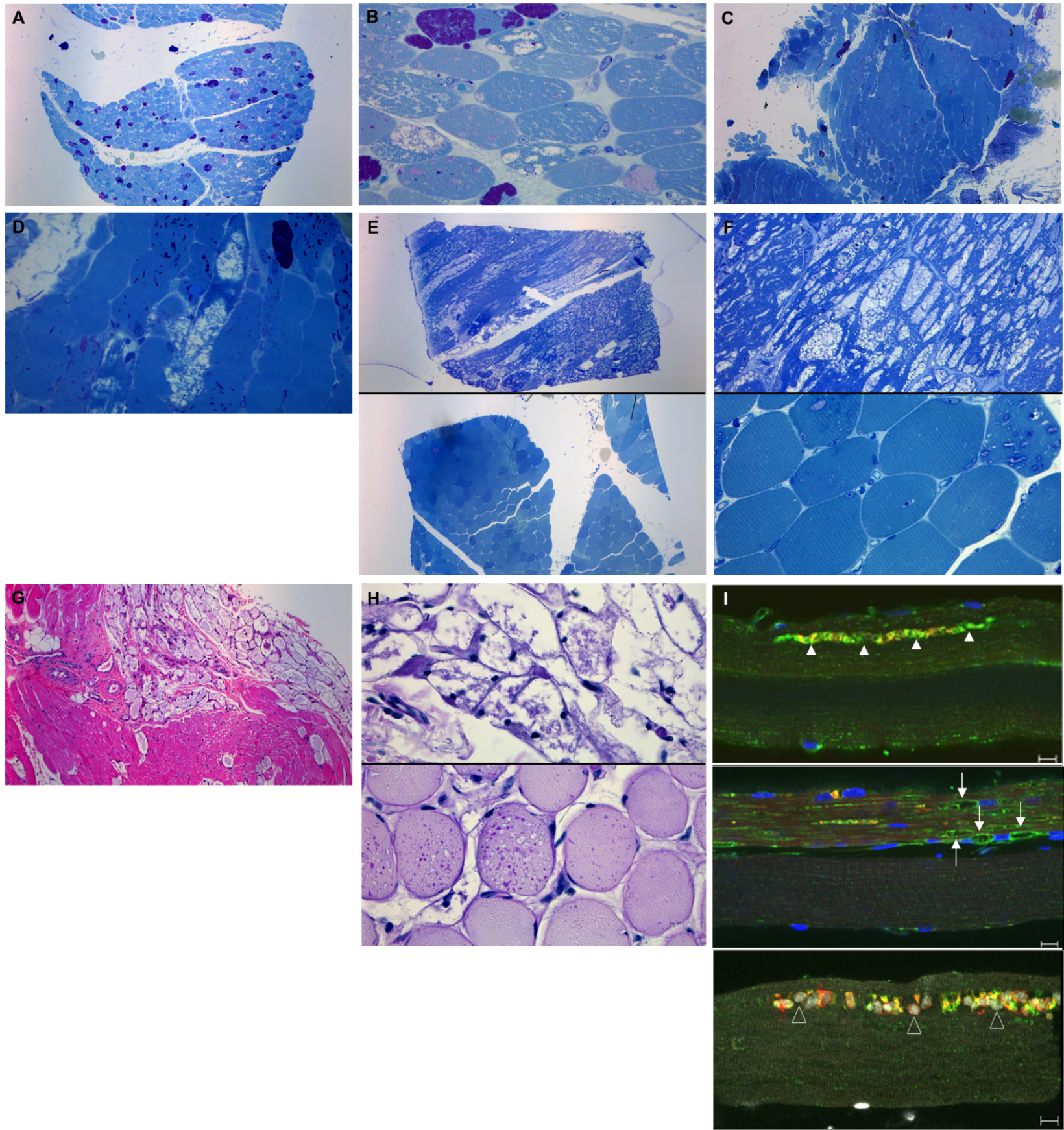

Supplement: Additional file 1: Figure S1 — Analysis of quadriceps muscle biopsy from Patient 2 prior to and following 12 and 96 months of ERT. (A) Epon-embedded PAS/Richardson’s-stained section shows mild to extensive vacuolation in many fibers (magnification 25×). (B) At a higher magnification (630×), it is evident that nearly all fibers are at least mildly affected. There is evidence of fibrosis and myophagocytosis. (C, D) After 12 months of ERT (age 19.0 months, or 17.6 months CGA), fewer fibers are effaced overall, and normal looking fibers can be seen (25× and 630×, respectively). (E) After 96 months of ERT (age 103.0 months, or 101.6 months CGA), some sections show regions with effaced or prominently vacuolated fibers (top), whereas other sections show mildly affected and normal looking fibers (bottom) (25×). Similar observations are noted at a higher magnification (F, top and bottom; 100× and 630×, respectively). The data are confirmed by H&E (G; 25×) and PASD (H, top and bottom; 630×) staining. There is little interstitial fibrosis even in the more affected areas (H, top; H, bottom: less affected area). (I) LAMP2/LC3 staining demonstrates prominent autophagic buildup (top fiber; arrowheads) in many fibers, rare fibers with large expanded lysosomes (middle; arrows), and fibers with autofluorescent inclusions (bottom, open arrowheads). Bar: 10 μm. [file 1750-1172-8-90-S1.docx]

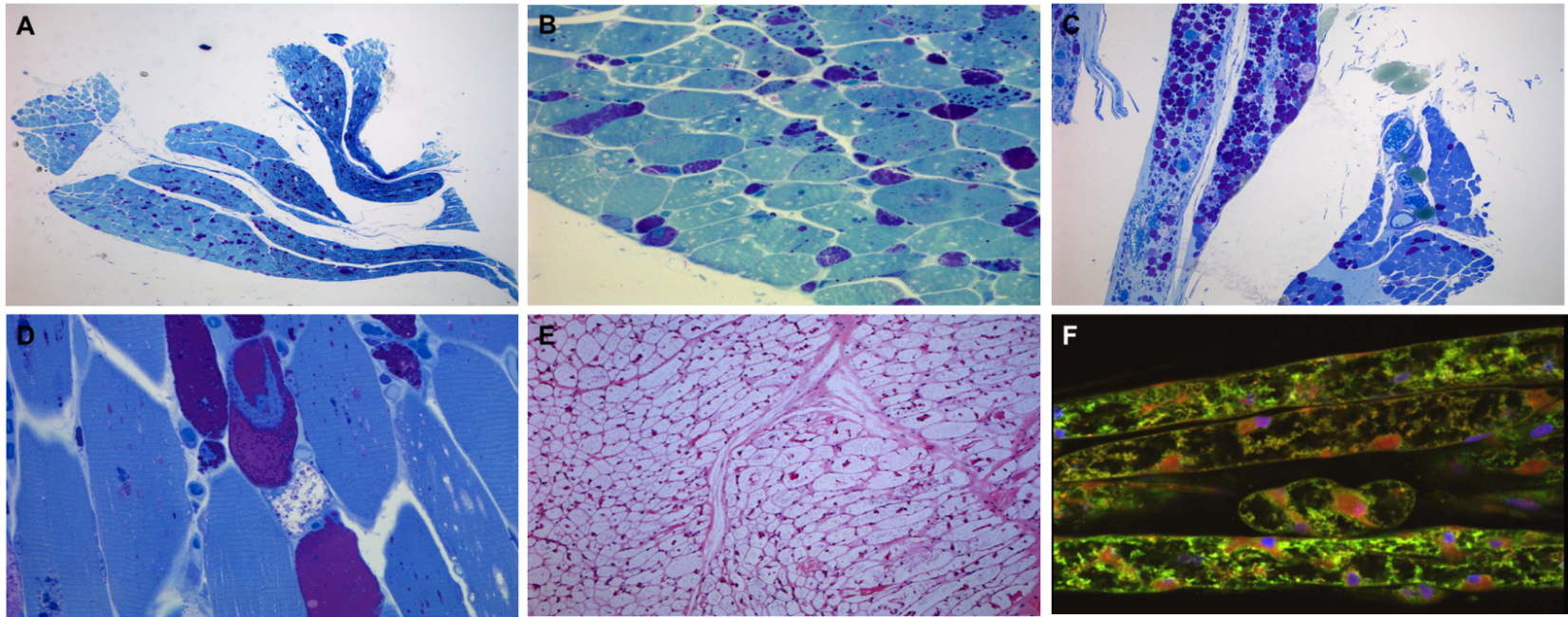

Supplement: Additional file 2: Figure S2 — Analysis of quadriceps muscle biopsy from Patient 3 prior to and following 12 and 85 months of ERT. (A) Epon-embedded toluidine blue/PAS-stained section shows mild to extensive vacuolation (magnification 25×). (B) At a higher magnification (100×), it is evident that nearly all fibers are at least mildly affected. There is minimal fibrosis. (C, D) After 12 months of ERT (age 17.4 months, or 16.9 months CGA), more fibers are effaced overall and there is severe fibrosis (C; 25×); autophagic debris (C) is detected at a higher magnification (D; circled; 630×). (E) After 85 months of ERT (age 90.4 months, or 89.9 months CGA), H&E-stained frozen sections show complete effacement of myofibrillar architecture (50×). (F) LAMP2/LC3 immunostaining demonstrates near‒complete destruction of muscle fibers. Bar: 10 μm. [file 1750-1172-8-90-S2.docx]

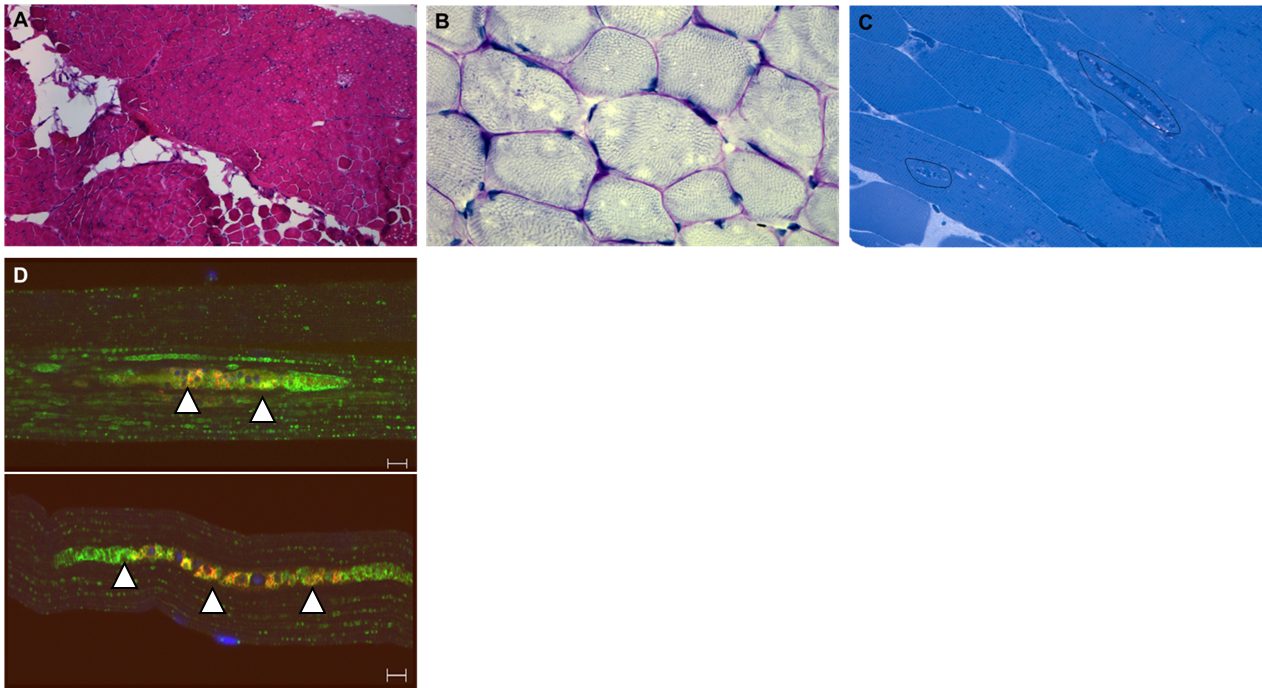

Supplement: Additional file 3: Figure S3 — Analysis of quadriceps muscle biopsy from Patient 4 after 77 months of ERT (age 77.5 months, or 77.2 months CGA). (A) H&E-stained frozen section shows the presence of vacuolated fibers in some fascicles (magnification 25×). Overall, muscle fibers are largely intact with limited evidence of regeneration (i.e., internal nuclei). (B) PAS-D staining demonstrates intact internal fiber architecture in most fibers, and no significant increase in interstitial stroma (630×). (C) Epon-embedded toluidine blue-stained section shows autophagic debris in mildly-affected fibers (circled; 630×). (D) LAMP2/LC3 immunostaining demonstrates largely intact muscle fibers (for example, top fiber in top panel) interspersed with moderately affected muscle fibers with prominent autophagic pathology (arrowheads). Bar: 10 μm. [file 1750-1172-8-90-S3.docx]

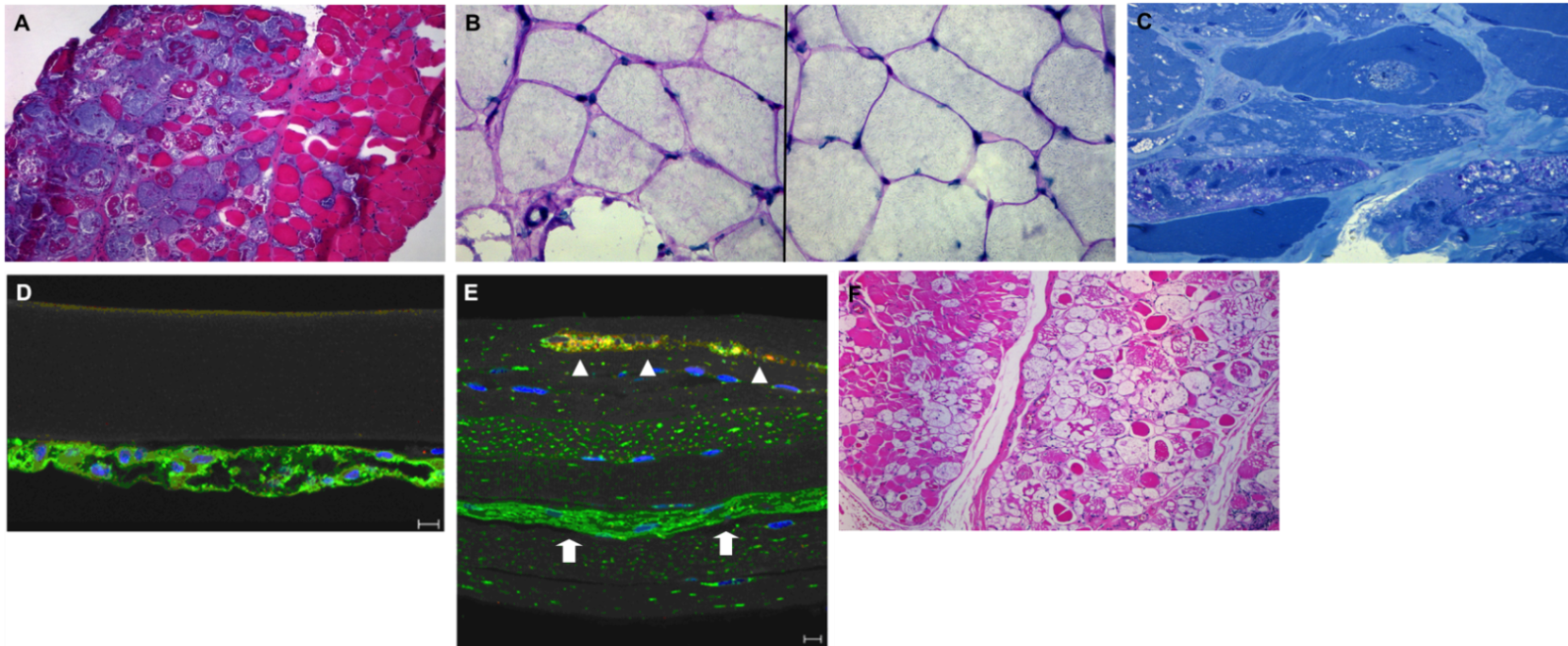

Supplement: Additional file 4: Figure S4 — Analysis of muscle biopsies from Patient 5 after 83 and 84 months of ERT. (A) H&E-stained frozen section shows the variation in pathology within a quadriceps biopsy: badly damaged fascicles are seen adjacent to largely intact ones; there is some evidence of regeneration (i.e., internal nuclei) (magnification 25×). (B, left and right) PAS-D staining demonstrates relatively intact fibers next to completely effaced ones; interstitial stroma are increased (630×). (C) Epon-embedded toluidine blue-stained section (630×) shows vacuolization in many fibers. (D, E) LAMP2/LC3 immunostaining demonstrates great variability of muscle fiber involvement in biopsies from both quadriceps (D) and SCM (E) muscles: a normal fiber (D; top fiber) next to a completely destroyed one (D; bottom fiber), a fiber with autophagic buildup (the buildup is seen in ~10 % of fibers) (E; arrowheads), and fibers with largely expanded lysosomes (E; arrows). Bar: 10 μm. (F; 25×) H&E-stained paraffin section of SCM muscle shows variation in pathology: severely damaged fascicles adjacent to less affected fascicles; there is some evidence of regeneration (i.e., internal nuclei); note the focal reactive mononuclear inflammation (lower right). Note: The images in A-D show tissue from the quadriceps biopsy taken after 84 months of ERT (age 86.9 months). The images in E and F show tissue from the SCM biopsy taken after 83 months of ERT (age 85.9 months). [file 1750-1172-8-90-S4.docx]

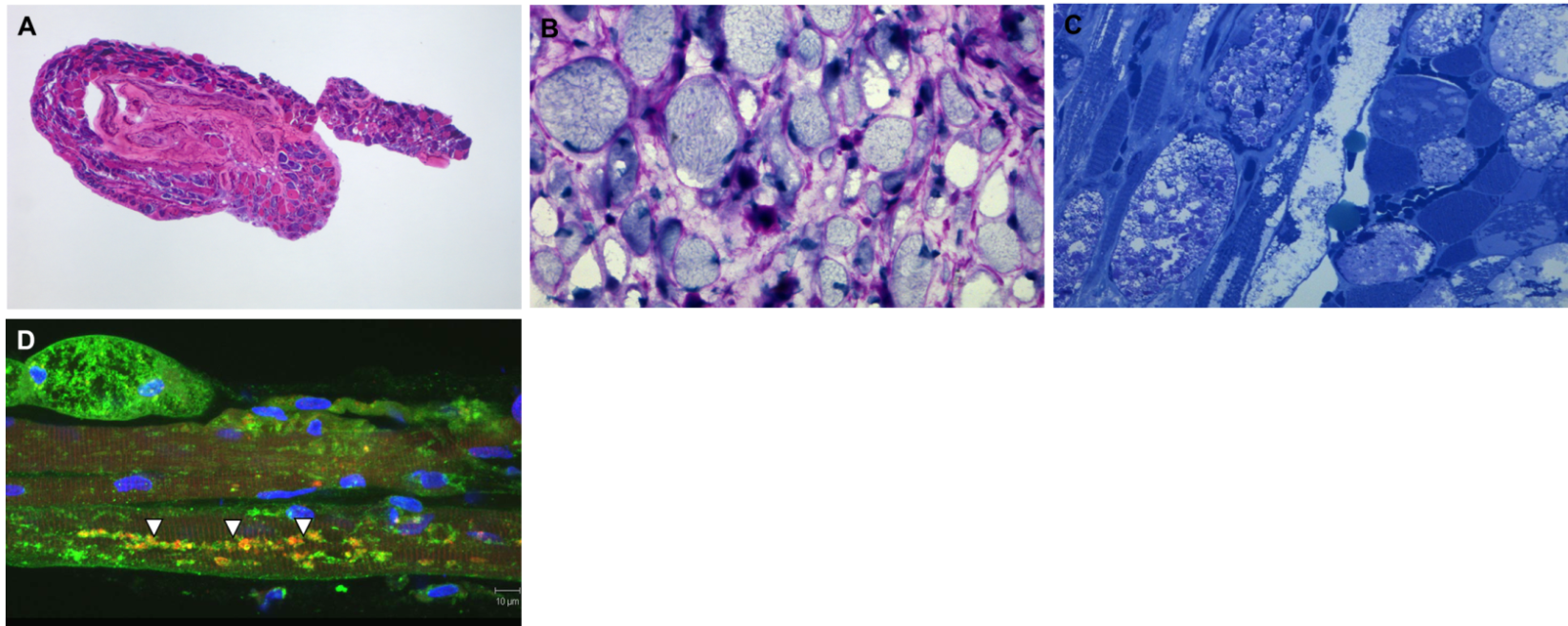

Supplement: Additional file 5: Figure S5 — Analysis of quadriceps muscle biopsy from Patient 6 after 49 months of ERT (age 56.0 months, or 55.0 months CGA). (A) H&E-stained frozen section shows the pattern of damage and fibrosis: vacuolization of more than 50% of myocytes, interspersed with fibers showing intact myofibrillar architecture and some internal nuclei indicating regeneration (magnification 25×). (B) PAS-D staining demonstrates relatively intact fibers in close proximity to completely effaced ones and increased interstitial stroma (630×). (C) Epon-embedded toluidine blue-stained section confirms the results obtained with H&E and PAS-D staining (630×). (D) LAMP2/LC3 immunostaining further demonstrates variability of muscle fiber involvement: a completely destroyed fiber (top fiber), a relatively preserved fiber with moderately enlarged lysosomes (middle fiber), and a fiber with autophagic accumulation (bottom fiber, arrowheads). Bar: 10 μm. [file 1750-1172-8-90-S5.docx]

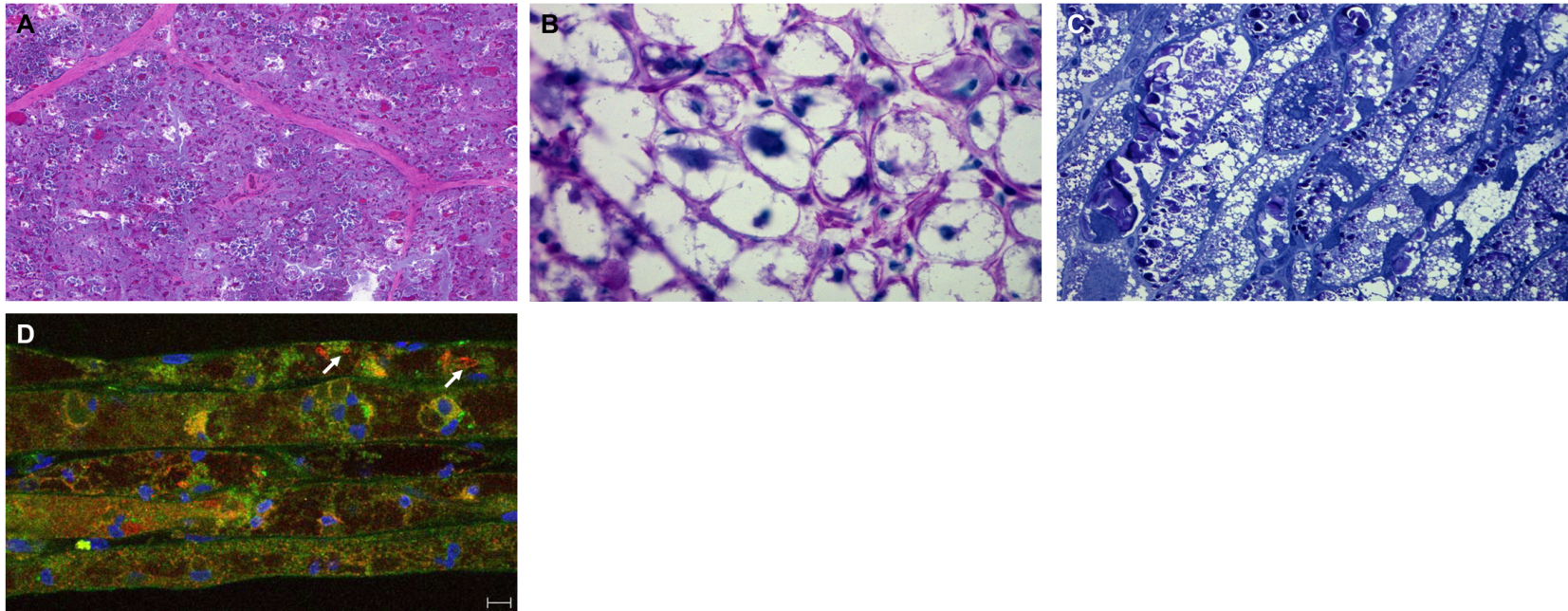

Supplement: Additional file 6: Figure S6 — Analysis of soleus muscle biopsy from Patient 8 after 18 months of ERT (age 24.0 months). (A) H&E-stained frozen section shows the extent of damage and fibrosis: severe, diffuse vacuolization of more than 95% of myocytes (magnification 25×). (B) PAS-D staining demonstrates loss of internal muscle architecture in virtually all fibers (only lace-like remnants can be seen); there is an increase in interstitial stroma (630×). (C) Epon-embedded toluidine blue-stained section highlights the obliteration of sarcoplasm by small vesicular structures (630×). (D) LAMP2/LC3 immunostaining demonstrates complete destruction of myofibers with a few identifiable autophagosomes (arrows) in the top fiber. Bar: 10 μm. [file 1750-1172-8-90-S6.docx]
